# Shot noise limited soft x-ray absorption spectroscopy in solution at a SASE-FEL using a transmission grating beam splitter

**DOI:** 10.1063/4.0000049

**Published:** 2021-01-29

**Authors:** Robin Y. Engel, Maria Ekimova, Piter S. Miedema, Carlo Kleine, Jan Ludwig, Miguel Ochmann, Benjamin Grimm-Lebsanft, Rory Ma, Melissa Teubner, Siarhei Dziarzhytski, Günter Brenner, Marie Kristin Czwalinna, Benedikt Rösner, Tae Kyu Kim, Christian David, Sonja Herres-Pawlis, Michael Rübhausen, Erik T. J. Nibbering, Nils Huse, Martin Beye

**Affiliations:** 1 Deutsches Elektronen Synchrotron DESY, 22607 Hamburg, Germany; 2 Max Born Institute for Nonlinear Optics and Short Pulse Spectroscopy, 12489 Berlin, Germany; 3 Institut for Nanostructure and Solid-State Physics, CFEL, University of Hamburg, 22761 Hamburg, Germany; 4 Institute of Inorganic Chemistry, RWTH Aachen University, 52074 Aachen, Germany; 5 Paul Scherrer Institute, 5232 Villigen PSI, Switzerland; 6 Department of Chemistry, Yonsei University, 03722 Seoul, South Korea

## Abstract

X-ray absorption near-edge structure (XANES) spectroscopy provides element specificity and is a powerful experimental method to probe local unoccupied electronic structures. In the soft x-ray regime, it is especially well suited for the study of 3*d*-metals and light elements such as nitrogen. Recent developments in vacuum-compatible liquid flat jets have facilitated soft x-ray transmission spectroscopy on molecules in solution, providing information on valence charge distributions of heteroatoms and metal centers. Here, we demonstrate XANES spectroscopy of molecules in solution at the nitrogen *K*-edge, performed at FLASH, the Free-Electron Laser (FEL) in Hamburg. A split-beam referencing scheme optimally characterizes the strong shot-to-shot fluctuations intrinsic to the process of self-amplified spontaneous emission on which most FELs are based. Due to this normalization, a sensitivity of 1% relative transmission change is achieved, limited by fundamental photon shot noise. The effective FEL bandwidth is increased by streaking the electron energy over the FEL pulse train to measure a wider spectral window without changing FEL parameters. We propose modifications to the experimental setup with the potential of improving the instrument sensitivity by two orders of magnitude, thereby exploiting the high peak fluence of FELs to enable unprecedented sensitivity for femtosecond XANES spectroscopy on liquids in the soft x-ray spectral region.

## I. INTRODUCTION

X-ray absorption near-edge structure (XANES) spectroscopy is one of the most common methods of x-ray spectroscopy, providing detailed information on local electronic structures: the position and magnitude of spectral features reports on oxidation states, spin configurations, and chemical bonds with element selectivity for light elements such as carbon, nitrogen, and oxygen as well as the important 3*d* transition metals.^1^ For the latter elements, the absorption lines in the soft x-ray regime are typically tenfold narrower than those in the hard x-ray regime, which enhances the spectral information content.^2^ Generally, the photochemistry of organic compounds and transition-metal complexes is of great interest for a variety of fields in chemistry and materials science, some examples being light harvesting in artificial photosynthesis and sensitizers for photovoltaic cells.^3–5^ Many important chemical processes occur in solution, where soft XANES spectroscopy can be used to specifically investigate the state and evolution of the electronic configuration and, thus, the molecular bonds involving a targeted element (ideally a light element or 3*d* transition metal) within the liquid phase. However, the solute typically constitutes only a small fraction of the sample ($\leq 10^{-3}$) compared to the large fraction of solvent molecules. Solvent absorption in the soft x-ray range is substantial and must be addressed both experimentally and in the data analysis, which is especially challenging at sources like free-electron lasers (FELs), where the strongly fluctuating incident flux also needs to be accounted for. The very short absorption lengths (1–10 *μ*m) severely limit the absolute amount of solute molecules that can be probed by the x-rays and require correspondingly thin samples in transmission measurements.

Early synchrotron-based static and time-resolved soft x-ray transmission studies in solution were conducted on stationary liquid targets^6–10^ (with the time resolution being typically around 100 ps, at slicing sources reaching below 200 fs). The development of stable, micron-thin, free-flowing liquid sheets (flat jets) in vacuum^11^ has enabled a more general application of time-resolved soft x-ray absorption spectroscopy in transmission.^12–17^ Recently, split single nozzles and gas dynamic nozzles have also been used to create such flat jets.^18,19^ The flow speeds of several 10 m/s (i.e., several 10 μm/μs) in these jets enable pump–probe experiments up to MHz repetition rates.

In this publication, we demonstrate the application of an advanced normalization scheme to soft x-ray spectroscopy in liquid solutions. We show transmission spectra of acetonitrile (ACN) and 4-aminoazobenzene (AAB) around the nitrogen *K*-edge, recorded from a micrometer thin free-flowing liquid sheet at the free-electron laser in Hamburg (FLASH) using a transmission grating-based beam-splitting scheme for shot-to-shot normalization. We demonstrate 95.4% confidence intervals (CIs) of ±5 mOD (about ±1% relative transmission change, see Fig. 3) within an acquisition time of 90 min. The referencing scheme reduced the noise level of our measurement setup to the fundamental limit of photon counting statistical noise. We further lay out potential improvements to the optical setup, which will increase the effective photon flux by up to four orders of magnitude, thus increasing the shot-noise limit on sensitivity by about two orders of magnitude for a similar measurement duration. With these improvements, we expect that such experiments at soft x-ray FELs with superconducting accelerators will enable unique sensitivity for ultrafast absorption studies.

## II. EXPERIMENTAL

**FIG. 1.**
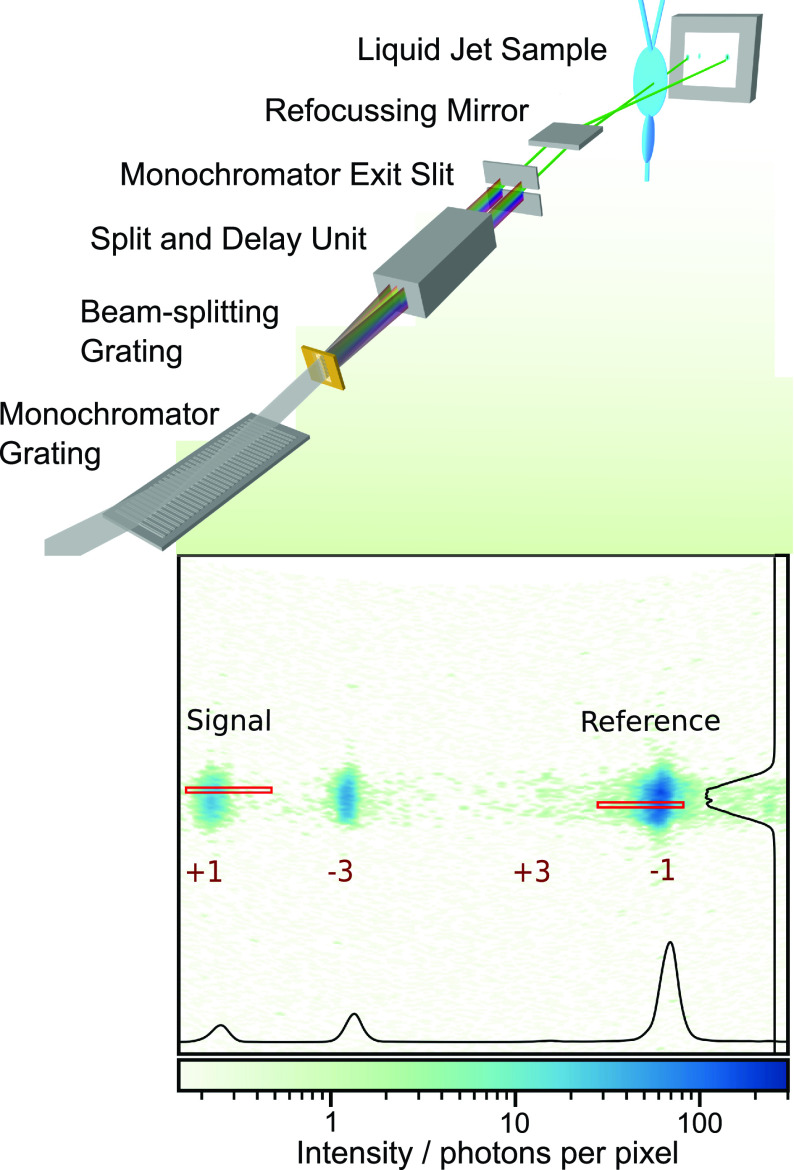
Experimental setup at the plane-grating monochromator beam line at FLASH. The incident beam is vertically dispersed by a reflection grating. A low line-density transmission grating splits the beam horizontally. A split-and-delay unit steers signal and reference beams toward the CCD detector, while only the signal beam interacts with the jet. The preprocessed detector image shows the first diffraction orders of the transmission grating (which constitute signal and reference beams) as well as weak contributions from the third orders that are partially clipped by apertures in the beam line. The black projections are drawn from an average image. Red boxes display the highly correlated regions used for normalization.

**FIG. 2.**
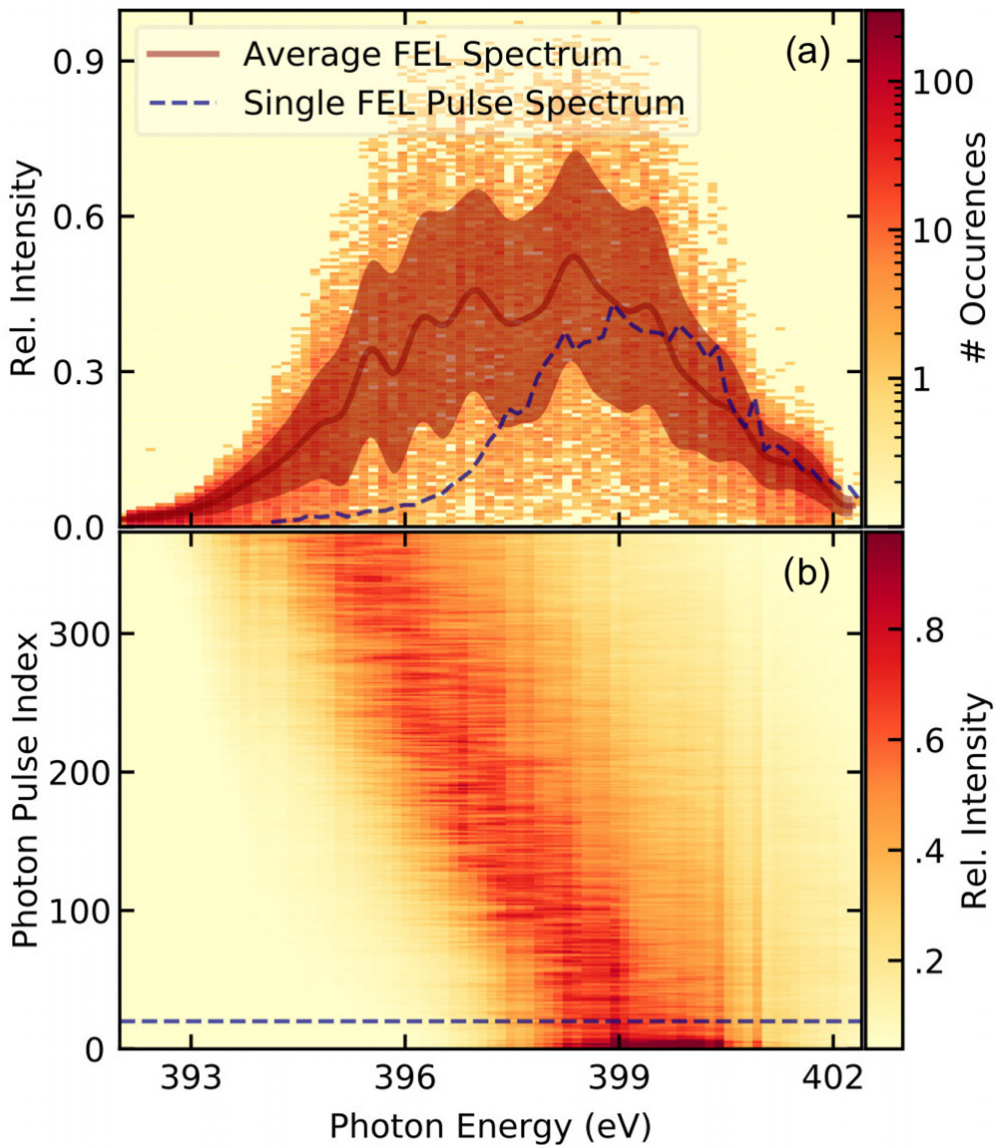
(a) Intensity distribution of the reference beam during the acquisition of the spectrum of 4-aminoazobenzene shown in Fig. 3. The overall distribution is broadened in comparison to the single pulse FEL spectrum due to the energy chirp over the bunch train. The average FEL spectrum is shown as a line, and the standard deviation is shaded. (b) The photon pulse-resolved spectra of a single bunch train, measured using the KALYPSO detector, demonstrating the chirp of the central photon energy across the bunch train. The dashed line indicates where the single pulse spectrum in (a) was extracted. Systematic gain variations of the detector have been corrected for, but artifacts from inhomogeneities of the used phosphor screen are still visible.

The measurements are performed at FLASH, producing x-ray bursts every 100 ms, each consisting of 400 pulses at a spacing of 1 *μ*s. The beam path is defined through two apertures of 1 mm diameter into the second branch of the plane-grating monochromator beamline PG2.^20,21^ The setup at the beam line is sketched in Fig. 1. The monochromator disperses the beam in the vertical direction and is tuned to the third-harmonic radiation with photon energies of around 400 eV, resonant to the *K*-edge absorption of nitrogen. During the measurements, the central transmitted photon energy of the monochromator is constantly scanned from 392.0 eV to 402.5 eV. The dispersed beam is directed onto a low line-density free-standing gold grating with a period of 11.3 *μ*m, a thickness of 140 nm, a width of 300 *μ*m, and a height of 4 mm, accepting the full height of the dispersed beam. This grating splits the FEL beam horizontally, with the zeroth diffraction order containing about 25% of the photons and two identical first orders containing about 10% of the photons each.^22^ The optics of the split-and-delay unit (SDU), permanently installed at the beam line,^23^ are used to separate the different orders, block the zeroth order beam, and steer the first-order beams (signal and reference) through the beam line. The photon energy is selected by the exit slit of the beam line (set to 200 *μ*m, transmitting an energy bandwidth of about 0.8 eV), and the beams are refocused onto the liquid flat jet in the experimental chamber. The operating conditions of the jet are as previously reported.^14^ One of the beams transmits centrally through the main leaf of the liquid flat jet, while the other bypasses the jet as a reference beam. Both beams are separated in the sample plane by about 1.7 mm. After transmission through a 200 nm thin aluminum filter for separating the jet chamber vacuum from the detector and blocking optical photons, both beams hit a charge-coupled device (CCD model ANDOR Ikon L936) cooled to about 210 K and installed about 1 m behind the sample. The detector is configured to read out the (13.5 *μ*m)^2^ square pixels with a binning of two by eight in the vertical and horizontal directions, respectively, generating images of 290 × 90 pixels. The pixel readout rate is set to 1 MHz, resulting in a usable frame rate of 3.3 Hz. The number of FEL bursts is accordingly reduced with a mechanical chopper.^24^ Each image read from the detector is accumulated over all 400 pulses in a burst. The CCD did not exceed 12% saturation. An averaged dark image is subtracted from each raw image. To enable a finer selection of regions of interest (ROI), the number of pixels is enhanced by cubic interpolation while conserving the value of the intensity integral. Due to the CCD being positioned behind the beamline focus, the exit slit image exhibits a curved distortion, which was characterized by a second-order polynomial fit to the exit slit shadow and corrected for by shifting columns of the image along the nondispersive direction such that the exit slit appears as a straight line, as visible in the corrected CCD image in Fig. 1. The routinely usable spectral range for such measurements is given by the FEL photon energy bandwidth. FELs operated in the based on Self-Amplified Spontaneous Emission (SASE) typically produce average bandwidths on the order of 1%,^25^ which are often narrower than the spectral range of interest. In order to change the central energy of FLASH1 (which uses fixed gap undulators), it is necessary to change the electron energy and, accordingly, the electron optics in the accelerator. Unfortunately, this often results in slight alterations of the photon beam path that can produce systematic deviations in the spectrum. Such artifacts are avoided by introducing a constant chirp into the electron energy across the burst, which produces a systematic shift of central photon energy evolving from the first to the last pulse in the train. This doubles the usable photon energy range for the experiment. In addition, the chirped bunch train provides a relatively flat intensity distribution across a large part of the photon energy window, as shown in Fig. 2(a). A direct measurement of the average photon energy variation across the burst is displayed in Fig. 2(b), where an acquisition from an online spectrometer^26^ equipped with the MHz line-detector KALYPSO^27^ is shown.

**FIG. 3.**
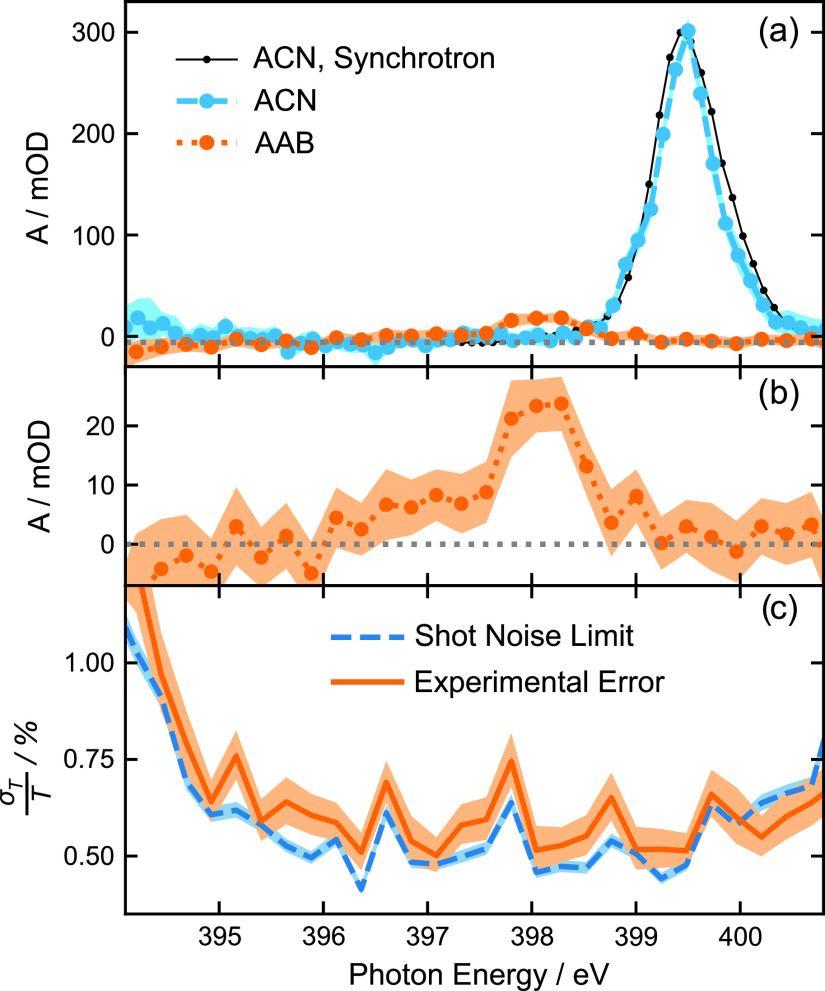
(a) Nitrogen *K*-edge transmission spectra of 1 M acetonitrile (ACN) and 40 mM 4-aminoazobenzene (AAB) in ethanol solution. A rescaled reference spectrum of 11.3 M ACN in water recorded at an undulator beamline of BESSY II with a resolution of 230 meV is shown for comparison. The ACN spectrum is calculated from 7600 images recorded within 46 min, and the AAB spectrum from 17 500 images is recorded over 88 min. The colored areas around the spectra mark the boundaries of 95.4% confidence intervals (CI) of the spectral absorbance. The absorbance values of 1.79 *μ*m and 1.71 *μ*m of ethanol as computed based on the CXRO database^28^ were subtracted from the ACN and AAB spectra, respectively. (b) Zoom in of the AAB spectrum. (c) The relative statistical uncertainty of the AAB measurement (68.3% CI of the transmittance divided by the transmittance) compared with the photon shot noise limit, i.e., the geometrically added square root of the estimated number of photons in both beams.

As a first assessment of this setup, XANES measurements are performed on 1 M ACN and 40 mM AAB ethanol solutions [see Figs. 3(a) and 3(b)]. Strong outliers in the intensity ratio ascribed to short-lived disturbances of the liquid jet are dropped from the analysis. The remaining images are sorted into bins corresponding to 120 meV for ACN or 240 meV intervals for the AAB solution. The recorded ACN spectrum is compared with a synchrotron spectrum, recorded from an 11.3 M solution in water as total fluorescence yield mode at the U41-PGM beam line of BESSY II with an energy resolution of 230 meV. Spectra are calibrated to the ACN resonance (1 s to 2 $\pi^{*}$) at 399.5 eV.

The split-beam referencing scheme works on the assumption that the signal and reference beams are identical in all aspects except for the interaction of the signal beam with the liquid jet. To properly extract correlated intensities of the two beams, the ROIs had to be carefully selected: a rotational misalignment of the fixed beam-splitting grating with respect to the dispersion direction of the monochromator resulted in different parts of the FEL spectrum reaching the CCD. To mitigate this issue, the monochromator exit slit was widened to 200 *μ*m, transmitting a spectral window of 0.8 eV. Although this compromises the energy resolution of the acquired spectra, the dispersion preserved on the CCD retained a resolution of around 0.5 eV, dominated by the out-of-focus blurring effect. More importantly, the widened exit slit allowed for certain regions on the CCD to detect the same part of the original FEL spectrum despite the misalignment of the beam splitting grating. Thus, the energy shift between the signal and reference beams could be compensated by proper selection of adequately small ROIs on the CCD, albeit at the cost of omitting roughly 87% of detected photons. The Pearson correlation coefficient (CC) between beam intensities was chosen as a measure to judge the quality of ROI selection. Near perfect correlations between 0.97 and 0.99 are reached over the flat regions of the spectra. The height of the ROIs is kept narrow at six pixels, while positions and the common width of the ROIs are optimized for the highest CC. The ROIs are selected sufficiently wide to cover the small position variations due to the chromaticity of the beam-splitting grating. A result is shown in Fig. 1. From the optimization, we determine a rotational misalignment of the beam-splitting grating of 0.8°. This correlation analysis works best on regions of flat spectral transmission, as variations of the sample absorption reduce the correlation^29^ between the signal and the reference. Therefore, the algorithm excludes images containing absorption peaks, namely, monochromator settings from 398.75 eV to 400.25 eV for ACN and from 377.3 eV to 398.7 eV for AAB. Images from outside the central range of the FEL bandwidth (393 eV–401 eV) are also excluded. A slow fluctuation in the background signal is drawn from an equally sized ROI within the dark region of the CCD, averaged over 30 consecutive images, and subtracted from all intensities.

The transmittance in each interval of the spectrum,

$$T=\langle I_{\mathrm{sig}}\rangle /\langle I_{\mathrm{ref}}\rangle ,$$ (1)

is then calculated as the ratio of averaged signal and reference intensities; absorbance A is the negative decadic logarithm of the transmittance,

$$A=-\mathrm{lo}g_{10}(T).$$ (2)

Finally, the absorbance of the ethanol solvent is subtracted, as calculated from the Center for x-Ray Optics (CXRO) database^28^ using the room temperature density of ethanol and the average jet thickness that best fits the baseline of the spectrum (1.79 *μ*m for the ACN measurement and 1.71 *μ*m for AAB). The ACN measurement consists of two, the AAB measurement of three combined datasets, for each of which the ROI optimization is performed individually. Error bars represent 95.4% confidence intervals (CI) for the expectation value of absorbance.

Since the intensities of the signal and the reference are strongly correlated, their statistical uncertainties (corresponding to 68.3% CI),

$$\sigma_{\mathrm{sig}}=\sqrt{\langle {\left(I_{\mathrm{sig}}-\langle I_{\mathrm{sig}}\rangle \right)}^{2}\rangle /N_{\mathrm{samples}}^{2}},$$ (3)

and *σ_ref_* (calculated correspondingly) are propagated with consideration of the covariance $\sigma_{I_{\mathrm{sig}}I_{\mathrm{ref}}}$ of both intensities for each bin, following the study by Tellinghuisen^30^ and the pertinent literature as

$$\sigma_{T}=\left|\frac{\left\langle I_{\mathrm{sig}}\right\rangle }{\left\langle I_{\mathrm{ref}}\right\rangle }\right|\sqrt{{\left(\frac{\sigma_{\mathrm{sig}}}{\left\langle I_{\mathrm{sig}}\right\rangle }\right)}^{2}+{\left(\frac{\sigma_{\mathrm{ref}}}{\left\langle I_{\mathrm{ref}}\right\rangle }\right)}^{2}-\frac{2\sigma_{I_{\mathrm{sig}}I_{\mathrm{ref}}}}{\left\langle I_{\mathrm{sig}}\rangle \langle I_{\mathrm{ref}}\right\rangle }}.$$ (4)

Figure 3(c) compares the measured relative statistical fluctuations $\sigma_{T}/T$ of the transmittance with the fluctuations that should be expected theoretically from a purely shot-noise limited process. The uncertainty of the absorbance *A* is

$$\sigma_{A}=\left|\frac{\sigma_{T}}{\mathrm{Tln}(10)}\right|,$$ (5)

which is doubled to attain the 95.4% CI.

Based on pixel-value histograms of the resulting images, the CCD reads an average of 31 (±2) analogs to digital units per 400 eV photon. Between central photon energies of 396 eV and 400 eV, we, thus, measure an effectively usable photon flux of 2.7 $\times 10^{3}$ photons per second in the reference beam ROI. Figure 3(c) shows that the determined experimental error is only slightly above the theoretical expectation for pure shot noise from the same number of photons. The measurements presented here are recorded within 1.5 h, mainly limited by the stability of the liquid jet conditions.

## III. DISCUSSION

XANES spectra of molecules and colloidal nanoparticles in solution are accessible either directly in transmission or by observing the partial or total fluorescence yield (PFY and TFY, respectively). Transmission and TFY detection modes are experimentally much simpler to implement as they do not require a spectrally resolved detection scheme. However, transmission measurements in solution determine sample absorption on a background of relatively large nonresonant solvent absorption, requiring thin liquid sheets and a precise normalization to the incident flux. In the case of TFY, the background from solvent fluorescence often dominates the overall signal. Still, TFY detection of XANES spectra in solution has been employed although this choice of detection scheme can lead to erroneous interpretations of the resulting spectra because solvent and sample fluorescence are competing processes that cannot be spectrally discriminated in this measurement mode.^31–34^ Therefore, TFY detection requires specific conditions, e.g., the solute absorption edges residing far below those of the solvent.^35^ On the other hand, partial fluorescence yield is a better measure of x-ray absorption; however, it is experimentally far more challenging, as it requires spectrally resolved detection that is selective to the elemental fluorescence of interest.^36^ Laser-driven high-harmonic generation (HHG) sources can provide time resolution in the femtosecond range and below,^47–50^ but the relatively low spectral photon flux at short wavelengths (<4 nm) still makes such XANES studies in solution challenging, albeit these sources hold great future potential.^37^ Free-electron lasers can, in principle, supply a much higher flux. However, most FELs are based on self-amplified spontaneous emission (SASE), intrinsically displaying strong spectral intensity fluctuations. These fluctuations have so far hampered quantitative soft XANES spectroscopy at these light sources, with only a few time-resolved studies published measuring partial and total fluorescence yield.^35,36,38–40^ The challenge to correctly normalize such fluctuations across a large dynamic range becomes especially severe for absorption measurements in transmission mode on dilute samples. Thus, recording solute spectra of millimolar concentrations on a background of 20–50 molar solvent density requires a high instrument sensitivity and, in turn, an excellent normalization to the incident flux. So far, no method of intensity normalization could be established as a standard in x-ray absorption measurements at FELs. Some facilities use Gas Monitor Detectors (GMDs)^41,42^ to measure the intensity fluctuations behind a monochromator. However, GMDs typically have a pulse-to-pulse accuracy of around 10% and struggle with the low pulse energy after a monochromator, which precludes their use for monochromatic third-harmonic radiation at FLASH. This challenge leads to the presented normalization scheme using a transmission grating beam splitter, utilizing a single CCD detector for incident and transmitted intensities. As this method can enable very high sensitivity, versions of it have been demonstrated at SPring-8 Angstrom Compact free electron LAser (SACLA)^43,44^ Linac Coherent Light Source (LCLS),^45^ and FLASH,^22,46^ showing considerably improved normalization of SASE fluctuations compared to previous normalization schemes. Implementing the same principle of split-beam normalization, we present the acquisition of XANES spectra from femtosecond FEL pulses with a sensitivity limited by photon counting statistics in the soft x-ray range on a liquid sample. High monochromatic x-ray flux in femtosecond pulses is currently only found at FELs and is especially beneficial in time-dependent studies of specific XANES features. We estimate an output of $7\times 10^{9}$ photons/s/0.1% bandwidth in the third harmonic at 400 eV produced by FLASH during this experiment based on the specified transmission of the beamline, monochromator, beam-splitting grating, and SDU. However, the number of detected photons is still low in comparison to other FEL measurements since this experiment utilized the third-harmonic radiation and mirrors that are optimized for the extreme ultraviolet wavelength range. For future studies, the soft x-ray monochromator beamline at FLASH 2 (under construction) offers higher flux and better beamline transmission for the third-harmonic radiation. The additional mirrors of the SDU used for individual beam steering and the alignment issue discussed above further attenuated the flux by nearly three orders of magnitude. The spectral flux that we could include in our analysis in this work reaches $5.5\times 10^{3}$ photons/s/eV due to the high repetition rate compared to other FEL studies. Some of these losses can be avoided in future studies as discussed below, with a potential increase by three to four orders of magnitude. Directly comparable published measurements were performed at HHG sources, which generally reach a much lower monochromatic flux: modern HHG setups can achieve tens of detected photons per second per eV, e.g., 14 photons/s/eV near the N *K*-edge in the study by Kleine *et al.*^37^ The spectral flux used by Obara *et al.*^44^ around the iron *K*-edge at 7.2 keV amounted to about $2.6\times 10^{4}$ photons/s/eV. On solid samples, Schlotter *et al.*^45^ report on average $6.4\times 10^{6}$ photons/s at the O *K*-edge, well in line with our work after the proposed improvements. For lower temporal resolutions, synchrotron studies offer a viable alternative with substantially more flux. Fondell *et al.*^12^ report $2.0\times 10^{9}$ photons/s/eV in a train of pulses of tens of picoseconds duration at the N *K*-edge at the BESSY II storage ring.

Many studies^37,43,44,46^ further exploit a dispersive acquisition scheme. Here, instead of scanning a monochromator and measuring the absorption for each step, the full spectrum of the source is dispersed onto a spatially resolving detector. Such an acquisition scheme can constitute an optimized parallel measurement utilizing the entire source bandwidth. If, as in this setup, beamline optics are used to disperse the beam and the sample is placed downstream, the sample is illuminated by a rainbow-like line focus, which necessarily couples spatial inhomogeneities into the spectrum. In the case of a liquid flat jet, the thickness often systematically varies in the vertical direction,^19^ requiring appropriate corrections. On the other hand, dispersion downstream of the sample^46^ allows for a small focus using the entire source bandwidth on the sample, provided that the sample can withstand the fluence of the full beam. Such a method then requires that one dimension of the detector is used for spectral resolution, so that the number of illuminated pixels per energy bin is substantially reduced. Thus, any detector inhomogeneities, nonlinearities, and digitization noise are not averaged out as well as in a monochromatic scanning scheme, where substantially larger areas of the detector can be utilized for each energy step. Building on the setup presented in Fig. 1, we propose the following adaptions for time-resolved future measurements to increase the detected flux and, thus, sensitivity:

First, we have already constructed a mount for the beam splitting grating, which allows fine-tuning the rotation angle, so that complications due to slight misalignments can be avoided in future measurements at FLASH. Furthermore, the transmission of the split-and-delay unit is optimized for longer wavelengths, but at the N *K*-edge, only 1% of the photons is transmitted. Thus, we will optimize the beam splitting grating design to directly generate the ideal beam separation in the sample plane, such that the use of the split-and-delay unit for beam steering is not required, resulting in two orders of magnitude higher effective flux. An additional step to maximize the effective photon flux can be taken by fully opening the exit slit of the monochromator and, thus, transitioning to a dispersive measurement. While this might amplify possible detector inhomogeneities and requires a normalization of varying sample thicknesses as discussed above, this imaging scheme would result in another order of magnitude increase in detected photon flux and provides a means to correct for slow drifts of the liquid jet thickness over time, which are, otherwise, likely to become the next significant source of error in long measurements with drastically reduced shot noise. Altogether, these improvements can increase the effective photon flux by up to four orders of magnitude, yielding more than 10^7^ detected photons per second in the reference beam. Similar quality spectra, as shown in Fig. 3, could ideally be recorded within approximately one second instead of more than one hour. For an hour-long measurement, the shot-noise limit on the sensitivity (scaling with the square root of the flux) would improve by a factor between 30 and 100 and, thus, reach less than ±100 *μ*OD.

## IV. CONCLUSION

In a setup designed for future time-resolved pump probe experiments, we measured x-ray absorption spectra of molecules in ethanol solution. We used the third-harmonic radiation of a SASE FEL and implemented a split-beam referencing scheme similar to those demonstrated at FELs in the hard x-ray regime and solid samples^22,43,45,46^ to compensate for intensity fluctuations, reaching 95.4% confidence intervals of ±5 mOD at the N *K*-edge. The sensitivity is close to the photon shot noise limit of this experiment. By introducing FELs to soft x-ray XANES spectroscopy on molecules in solution, the presented measurements are about eight times more sensitive than those previously achieved in femtosecond XANES on liquid solutions at this photon energy using an HHG source.^37^ In the presented measurements, with the sensitivity down to $\sigma_{T}/T≃0.5$, % relative transmission change (see Fig. 2) is comparable to the noise ratio of 0.26% achieved in a comparable setup in the hard x-ray regime.^43^

An alignment error and the use of the split-and-delay unit for beam steering reduced the effective photon flux. In order to better exploit the high photon flux of FLASH, we suggest an advanced configuration that fully omits both the SDU and the exit slit, thus gaining three to four orders of magnitude in intensity. As a consequence, we see the potential for acquiring spectra with mOD sensitivity within seconds, while the shot-noise limit on the 95.4% confidence intervals could be reduced to less than ±100 μOD within one hour. This constitutes an important step to enable measurements on the photoinduced dynamics of increasingly larger, less soluble molecules on the femtosecond timescale.

## AUTHORS' CONTRIBUTIONS

R.Y.E. and M.E. contributed equally to this work.

## Data Availability

The data that support the findings of this study are available from the corresponding author upon reasonable request.

## References

[1] J. Stöhr , *NEXAFS Spectroscopy*, Springer Series in Surface Sciences ( Springer-Verlag Belin Heidelberg GmbH, 1992), Vol. 25.

[2] F. J. Himpsel , Phys. Status Solidi B 248, 292 (2011). 10.1002/pssb.201046212

[3] I. Roger , M. A. Shipman , and M. D. Symes , Nat. Rev. Chem. 1, 1 (2017). 10.1038/s41570-016-0003

[4] X. Du , X. Jiao , S. Rechberger , J. D. Perea , M. Meyer , N. Kazerouni , E. Spiecker , H. Ade , C. J. Brabec , R. H. Fink , and T. Ameri , Macromolecules 50, 2415 (2017). 10.1021/acs.macromol.6b02699

[5] C. Xie , T. Heumüller , W. Gruber , X. Tang , A. Classen , I. Schuldes , M. Bidwell , A. Späth , R. H. Fink , T. Unruh , I. McCulloch , N. Li , and C. J. Brabec , Nat. Commun. 9, 1 (2018). 10.1038/s41467-018-07807-5 30559396 PMC6297219

[6] B. Yang and J. Kirz , Phys. Rev. B 36, 1361 (1987). 10.1103/PhysRevB.36.1361 9942965

[7] P. Wernet , G. Gavrila , K. Godehusen , C. Weniger , E. T. Nibbering , T. Elsaesser , and W. Eberhardt , Appl. Phys. A 92, 511 (2008). 10.1007/s00339-008-4726-5

[8] N. Huse , H. Wen , D. Nordlund , E. Szilagyi , D. Daranciang , T. A. Miller , A. Nilsson , R. W. Schoenlein , and A. M. Lindenberg , Phys. Chem. Chem. Phys. 11, 3951 (2009). 10.1039/b822210j 19440624

[9] H. Wen , N. Huse , R. W. Schoenlein , and A. M. Lindenberg , J. Chem. Phys. 131, 234505 (2009). 10.1063/1.3273204 20025333

[10] K. Hong , H. Cho , R. W. Schoenlein , T. K. Kim , and N. Huse , Acc. Chem. Res. 48, 2957 (2015). 10.1021/acs.accounts.5b00154 26488127

[11] M. Ekimova , W. Quevedo , M. Faube , P. Wernet , and E. T. Nibbering , Struct. Dyn. 2, 054301 (2015). 10.1063/1.4928715 26798824 PMC4711648

[12] M. Fondell , S. Eckert , R. M. Jay , C. Weniger , W. Quevedo , J. Niskanen , B. Kennedy , F. Sorgenfrei , D. Schick , E. Giangrisostomi , R. Ovsyannikov , K. Adamczyk , N. Huse , P. Wernet , R. Mitzner , and A. Föhlisch , Struct. Dyn. 4, 054902 (2017). 10.1063/1.4993755 28852689 PMC5555770

[13] M. Ekimova , W. Quevedo , L. Szyc , M. Iannuzzi , P. Wernet , M. Odelius , and E. T. Nibbering , J. Am. Chem. Soc. 139, 12773 (2017). 10.1021/jacs.7b07207 28810120

[14] M. Ekimova , M. Kubin , M. Ochmann , J. Ludwig , N. Huse , P. Wernet , M. Odelius , and E. T. Nibbering , J. Phys. Chem. B 122, 7737 (2018). 10.1021/acs.jpcb.8b05424 30024171

[15] S. Eckert , J. Norell , R. M. Jay , M. Fondell , R. Mitzner , M. Odelius , and A. Föhlisch , Chemistry 25, 1733 (2019). 10.1002/chem.201804166 30452789 PMC6470867

[16] R. M. Jay , S. Eckert , V. Vaz da Cruz , M. Fondell , R. Mitzner , and A. Föhlisch , Angew. Chem.-Int. Ed. 58, 10742 (2019). 10.1002/anie.201904761 PMC6771958 31145507

[17] L. Kjellsson , K. D. Nanda , J.-E. Rubensson , G. Doumy , S. H. Southworth , P. J. Ho , A. M. March , A. Al Haddad , Y. Kumagai , M.-F. Tu , R. D. Schaller , T. Debnath , M. S. Bin Mohd Yusof , C. Arnold , W. F. Schlotter , S. Moeller , G. Coslovich , J. D. Koralek , M. P. Minitti , M. L. Vidal , M. Simon , R. Santra , Z.-H. Loh , S. Coriani , A. I. Krylov , and L. Young , Phys. Rev. Lett. 124, 236001 (2020). 10.1103/PhysRevLett.124.236001 32603165

[18] G. Galinis , J. Strucka , J. C. Barnard , A. Braun , R. A. Smith , and J. P. Marangos , Rev. Sci. Instrum. 88, 083117 (2017). 10.1063/1.4990130 28863712

[19] J. D. Koralek , J. B. Kim , P. Bruza , C. B. Curry , Z. Chen , H. A. Bechtel , A. A. Cordones , P. Sperling , S. Toleikis , J. F. Kern , S. P. Moeller , S. H. Glenzer , and D. P. DePonte , Nat. Commun. 9, 1 (2018). 10.1038/s41467-018-05365-4 29636445 PMC5893585

[20] N. Gerasimova , S. Dziarzhytski , and J. Feldhaus , J. Mod. Opt. 58, 1480 (2011). 10.1080/09500340.2011.588344

[21] M. Wellhöfer , W. Wurth , and M. Richter , “ The monochromator beamline at FLASH assembly, characterization and first experiments,” Ph.D. thesis ( Hamburg University, 2007).

[22] G. Brenner , S. Dziarzhytski , P. Miedema , B. Rösner , C. David , and M. Beye , Opt. Lett. 44, 2157 (2019). 10.1364/OL.44.002157 31042172

[23] F. Sorgenfrei , W. F. Schlotter , T. Beeck , M. Nagasono , S. Gieschen , H. Meyer , A. Föhlisch , M. Beye , and W. Wurth , Rev. Sci. Instrum. 81, 043107 (2010). 10.1063/1.3374166 20441325

[24] E. Ploenjes and K. Tiedtke , in *Optical Technologies for Extreme-Ultraviolet and Soft X-Ray Coherent Sources*, Springer Series in Optical Sciences Vol. 197, edited by Canova F. and Poletto L. ( Springer, Heidelberg/Hamburg, 2015), Chap. 1, pp. 1–22.

[25] E. L. Saldin , E. A. Schneidmiller , and M. V. Yurkov , Opt. Commun. 148, 383 (1998). 10.1016/S0030-4018(97)00670-6

[26] G. Brenner , S. Kapitzki , M. Kuhlmann , E. Ploenjes , T. Noll , F. Siewert , R. Treusch , K. Tiedtke , R. Reininger , M. D. Roper , M. A. Bowler , F. M. Quinn , and J. Feldhaus , Nucl. Instrum. Methods Phys. Res., Sect. A 635, 99 (2011). 10.1016/j.nima.2010.09.134

[27] C. Gerth , G. Brenner , M. Caselle , S. Düsterer , D. Haack , D. Makowski , A. Mielczarek , S. Palutke , L. Rota , V. Rybnikov , C. Schmidt , B. Steffen , and K. Tiedtke , J. Synchrotron Radiat. 26, 1514 (2019). 10.1107/S1600577519007835 31490139 PMC6730618

[28] B. Henke , E. Gullikson , and J. Davis , At. Data Nucl. Data Tables 54, 181 (1993). 10.1006/adnd.1993.1013

[29] S. Zohar and J. J. Turner , Opt. Lett. 44, 243 (2019). 10.1364/OL.44.000243 30644871

[30] J. Tellinghuisen , J. Phys. Chem. A 105, 3917 (2001). 10.1021/jp003484u

[31] F. M. De Groot , Nat. Chem. 4, 766 (2012). 10.1038/nchem.1431 23000979

[32] T. Z. Regier , A. J. Achkar , D. Peak , J. S. Tse , and D. G. Hawthorn , Nat. Chem. 4, 765 (2012). 10.1038/nchem.1430 23000978

[33] P. S. Miedema , P. Wernet , and A. Föhlisch , Phys. Rev. A 89, 1 (2014). 10.1103/PhysRevA.89.052507

[34] P. S. Miedema and M. Beye , J. Phys. Chem. Lett. 9, 2579 (2018). 10.1021/acs.jpclett.8b00720 29715037

[35] Z. H. Loh , G. Doumy , C. Arnold , L. Kjellsson , S. H. Southworth , A. Al Haddad , Y. Kumagai , M. F. Tu , P. J. Ho , A. M. March , R. D. Schaller , M. S. Bin Mohd Yusof , T. Debnath , M. Simon , R. Welsch , L. Inhester , K. Khalili , K. Nanda , A. I. Krylov , S. Moeller , G. Coslovich , J. Koralek , M. P. Minitti , W. F. Schlotter , J. E. Rubensson , R. Santra , and L. Young , Science 367, 179 (2020). 10.1126/science.aaz4740 31919219

[36] R. Mitzner , J. Rehanek , J. Kern , S. Gul , J. Hattne , T. Taguchi , R. Alonso-Mori , R. Tran , C. Weniger , H. Schröder , W. Quevedo , H. Laksmono , R. G. Sierra , G. Han , B. Lassalle-Kaiser , S. Koroidov , K. Kubicek , S. Schreck , K. Kunnus , M. Brzhezinskaya , A. Firsov , M. P. Minitti , J. J. Turner , S. Moeller , N. K. Sauter , M. J. Bogan , D. Nordlund , W. F. Schlotter , J. Messinger , A. Borovik , S. Techert , F. M. De Groot , A. Föhlisch , A. Erko , U. Bergmann , V. K. Yachandra , P. Wernet , and J. Yano , J. Phys. Chem. Lett. 4, 3641 (2013). 10.1021/jz401837f 24466387 PMC3901369

[37] C. Kleine , M. Ekimova , G. Goldsztejn , S. Raabe , C. Strüber , J. Ludwig , S. Yarlagadda , S. Eisebitt , M. J. Vrakking , T. Elsaesser , E. T. Nibbering , and A. Rouzée , J. Phys. Chem. Lett. 10, 52 (2019). 10.1021/acs.jpclett.8b03420 30547598

[38] P. Wernet , K. Kunnus , I. Josefsson , I. Rajkovic , W. Quevedo , M. Beye , S. Schreck , S. Grübel , M. Scholz , D. Nordlund , W. Zhang , R. W. Hartsock , W. F. Schlotter , J. J. Turner , B. Kennedy , F. Hennies , F. M. De Groot , K. J. Gaffney , S. Techert , M. Odelius , and A. Föhlisch , Nature 520, 78 (2015). 10.1038/nature14296 25832405

[39] S. Eckert , J. Norell , P. S. Miedema , M. Beye , M. Fondell , W. Quevedo , B. Kennedy , M. Hantschmann , A. Pietzsch , B. E. Van Kuiken , M. Ross , M. P. Minitti , S. P. Moeller , W. F. Schlotter , M. Khalil , M. Odelius , and A. Föhlisch , Angew. Chem.-Int. Ed. 56, 6088 (2017). 10.1002/anie.201700239 PMC5485001 28374523

[40] R. M. Jay , J. Norell , S. Eckert , M. Hantschmann , M. Beye , B. Kennedy , W. Quevedo , W. F. Schlotter , G. L. Dakovski , M. P. Minitti , M. C. Hoffmann , A. Mitra , S. P. Moeller , D. Nordlund , W. Zhang , H. W. Liang , K. Kunnus , K. Kubiček , S. A. Techert , M. Lundberg , P. Wernet , K. Gaffney , M. Odelius , and A. Föhlisch , J. Phys. Chem. Lett. 9, 3538 (2018). 10.1021/acs.jpclett.8b01429 29888918

[41] K. Tiedtke , J. Feldhaus , U. Hahn , U. Jastrow , T. Nunez , T. Tschentscher , S. V. Bobashev , A. A. Sorokin , J. B. Hastings , S. Möller , L. Cibik , A. Gottwald , A. Hoehl , U. Kroth , M. Krumrey , H. Schöppe , G. Ulm , and M. Richter , J. Appl. Phys. 103, 094511 (2008). 10.1063/1.2913328

[42] A. A. Sorokin , Y. Bican , S. Bonfigt , M. Brachmanski , M. Braune , U. F. Jastrow , A. Gottwald , H. Kaser , M. Richter , and K. Tiedtke , J. Synchrotron Radiat. 26, 1092 (2019). 10.1107/S1600577519005174 31274432 PMC6613123

[43] T. Katayama , Y. Inubushi , Y. Obara , T. Sato , T. Togashi , K. Tono , T. Hatsui , T. Kameshima , A. Bhattacharya , Y. Ogi , N. Kurahashi , K. Misawa , T. Suzuki , and M. Yabashi , Appl. Phys. Lett. 103, 131105 (2013). 10.1063/1.4821108

[44] Y. Obara , T. Katayama , Y. Ogi , T. Suzuki , N. Kurahashi , S. Karashima , Y. Chiba , Y. Isokawa , T. Togashi , Y. Inubushi , M. Yabashi , T. Suzuki , and K. Misawa , Opt. Express 22, 1105 (2014). 10.1364/OE.22.001105 24515070

[45] W. F. Schlotter , M. Beye , S. Zohar , G. Coslovich , G. L. Dakovski , M. F. Lin , Y. Liu , A. Reid , S. Stubbs , P. Walter , K. Nakahara , P. Hart , P. S. Miedema , L. LeGuyader , K. Hofhuis , P. T. P. Le , J. E. t Elshof , H. Hilgenkamp , G. Koster , X. H. Verbeek , S. Smit , M. S. Golden , H. A. Durr , and A. Sakdinawat , arXiv:2006.13968 (2020).

[46] R. Engel , P. Miedema , D. Turenne , I. Vaskivskyi , G. Brenner , S. Dziarzhytski , M. Kuhlmann , J. Schunck , F. Döring , A. Styervoyedov , S. Parkin , C. David , C. Schüßler-Langeheine , H. Dürr , and M. Beye , Appl. Sci. 10, 6947 (2020). 10.3390/app10196947

[47] S. L. Cousin, N. Di Palo, B. Buades, S. M. Teichmann, M. Reduzzi, M. Devetta, A. Kheifets, G. Sansone, and J. Biegert, Phys. Rev. X 7, 041030 (2017). 10.1103/PhysRevX.7.041030

[48] A. S. Johnson, D. R. Austin, D. A. Wood, Christian Brahms, Andrew Gregory, K. B. Holzner, S. Jarosch, E. W. Larsen, S. Parker, C. S. Strüber, P. Ye, J. W. G. Tisch, and J. P. Marangos, Sci. Adv. 4, eaar3761 (2018). 10.1126/sciadv.aar3761 29756033 PMC5947981

[49] A. D. Smith, T. Balčiūnas, Y.-P. Chang, C. Schmidt, K. Zinchenko, F. B. Nunes, E. Rossi, V. Svoboda, Z. Yin, J.-P. Wolf, and H. J. Wörner, J. Phys. Chem. Lett. 11, 1981 (2020). 10.1021/acs.jpclett.9b03559 32073862 PMC7086398

[50] L. Barreau, A. D. Ross, S. Garg, P. M. Kraus, D. M. Neumark, and Stephen R. Leone, Sci. Rep. 10, 5773 (2020). 10.1038/s41598-020-62461-6 32238820 PMC7113301

